# The perceived fit of guided iCBT in specialised mental healthcare services: a qualitative study of healthcare professionals’ perspectives

**DOI:** 10.3389/fdgth.2026.1955151

**Published:** 2026-09-09

**Authors:** Beate Standal, Robin Maria Francisca Kenter, Monika Knudsen Gullslett, Tine Nordgreen, Inger Lise Teig

**Affiliations:** 1 Department of Global Public Health and Primary Care, Faculty of Medicine, University of Bergen, Bergen, Norway; 2 Research Centre for Digital Mental Health Services, Division of Psychiatry, Haukeland University Hospital, Bergen, Norway; 3 Norwegian Centre for E-health Research, University Hospital of Northern Norway, Tromsø, Norway

**Keywords:** digital mental health, e-health, healthcare professional, implementation, internet-delivered cognitive behavioural therapy iCBT, mental health, qualitative study, telehealth

## Abstract

**Background:**

Therapist-guided internet-delivered cognitive behavioural therapy (guided iCBT) is an effective treatment for common mental disorders and has the potential to improve access to care. However, its integration into routine practice remains limited. This study explored healthcare professionals’ perceptions of the suitability of guided iCBT in specialised mental healthcare and examined how perceived fit influences implementation across clinical and organisational contexts.

**Methods:**

Semi-structured interviews and participatory observations were conducted at three specialised mental health clinics in Norway that offered guided iCBT to adults with anxiety and depression. Thirty-one healthcare professionals were recruited through purposive and snowball sampling. Data were analysed using a combination of inductive and deductive approaches. The deductive analysis was informed by Lau et al.'s framework of contextual fit, which conceptualises fit as alignment between an intervention and professional, organisational, and external contexts.

**Results:**

Healthcare professionals’ perceptions of fit varied across contextual levels and influenced the implementation of guided iCBT. At the intervention level, participants identified a perceived mismatch between the evidence base for guided iCBT and limitations in its technological design, which was viewed as insufficiently aligned with contemporary therapeutic practices. At the professional level, differing professional values and therapeutic norms shaped perceptions of appropriateness and acceptability. At the organisational level, limited readiness for implementation and concerns about patient complexity in specialised care settings contributed to doubts about the intervention's suitability. At the external level, tensions emerged between evidence-based expectations and prevailing clinical assumptions favouring face-to-face care, further affecting perceptions of fit and legitimacy.

**Conclusions:**

The findings suggest that implementing guided iCBT in specialised mental healthcare depends on aligning the intervention with the clinical, organisational, and broader system contexts in which it is introduced. Efforts to improve technological usability, strengthen organisational readiness, engage clinicians, and address assumptions about legitimate forms of therapy may enhance perceived fit and support sustained implementation of guided iCBT.

## Background

Several studies have shown that therapist-guided internet-delivered cognitive behavioural therapy (guided iCBT) is an effective method for treating various mental health problems (1–3) without compromising the therapeutic alliance (4–6) and presents a promising solution to enhance access to treatment for common mental disorders (7, 8). The use of guided iCBT may reduce the demand for therapists (9), who are a scarce resource (10, 11). Despite reported challenges, including higher dropout rates compared to face-to-face therapy (12, 13), concerns about digital exclusion (14), issues related to equity (15), legal and ethical considerations (16) and therapists’ lack of competencies in this new modality (17), the advantages of guided iCBT are considered substantial. These advantages include improved access to evidence-based treatment, greater flexibility for patients, increased geographical reach, reduced waiting times, and more efficient use of limited therapist resources (7–9). Consequently, guided iCBT is recommended in the clinical guidelines of numerous countries (18–21). Guided iCBT has been available on a small scale for over two decades (22) and is gradually being implemented into regular treatment services (23). Despite its potential and long-standing presence, the uptake of guided iCBT in routine healthcare remains low (24, 25). This challenge will be explored in this paper, using the concept of “fit” to better understand how guided iCBT aligns—or fails to align—with various contextual factors influencing its implementation.

Extensive research has examined why evidence-based methods such as guided iCBT are underused in clinical practice (26), often focusing on barriers and facilitators to implementation (27). While some of these are concrete and systemic, others may reflect how individuals interpret their experiences (28). Understanding uptake thus requires attention to both perceived and actual factors. However, studies framed by the barriers-and-facilitators approach have been criticised for lacking critical reflexivity and for failing to adequately capture the complex, context-dependent nature of implementation challenges (27). As a result, scholars increasingly call for broader analytical frameworks (27, 29), such as realist evaluation, systems thinking, or process-oriented theories, to generate deeper, more actionable insights.

This evolving perspective highlights the growing recognition of contextual factors in implementation (30). Some scholars have also acknowledged the dynamic nature of context (31). For an intervention to be successfully implemented, it must fit the context in which it is applied (32). The concept of “fit” is prevalent across various implementation frameworks, such as the Consolidated Framework for Implementation Research (CFIR) (33), Exploration Preparation Implementation Sustainment (EPIS) (34), the Stetler Model (35), Promoting Action on Research Implementation in Health Services (PARISH) (36) and the Quality Implementation Framework (QIF) (37), where it serves as both a determinant of implementation and an implementation outcome. The concept of “fit” offers a valuable lens for interpreting several of Proctor et al.'s (38) widely cited implementation outcomes (39), particularly appropriateness (perceived fit), feasibility (actual fit), and acceptability (fit with individual values and identity). Some research has also addressed aspects of fit, including in digital mental health contexts (40–51), yet these insights remain fragmented and under-integrated into broader implementation discourse. Although fit is addressed in many frameworks and studies, often implicitly or under different labels, it remains fragmented and not fully developed as a theoretical lens in its own right (32).

Several implementation frameworks could have informed the present study. For example, CFIR provides a comprehensive taxonomy of implementation determinants, EPIS focuses on implementation processes across different phases, and Normalization Process Theory (NPT) explains how new practices become embedded in routine work (33, 34, 52). However, because the aim was to explore healthcare professionals’ perceptions of how guided iCBT fits clinical, organisational, and wider service contexts, Lau et al.'s (53) framework was selected. Unlike broader determinant or process-oriented frameworks, it explicitly centres on the relationship between an intervention and its context. This focus was particularly relevant for guided iCBT, where questions of suitability, legitimacy, and contextual alignment were central to the research question.

Building on Lau et al.'s (53) conceptualisation of implementation as dependent on the fit between interventions and multiple contextual levels, this study examines how healthcare professionals perceive the suitability of guided internet-delivered cognitive behavioural therapy (iCBT) within specialised mental healthcare services and how these perceptions influence implementation. Lau et al.'s framework offers useful insights into the evolving nature of contextual factors and their impact on implementation. Drawing on qualitative data from interviews and observations, the study seeks to understand healthcare professionals’ perceptions of the suitability of guided iCBT in their clinical and organisational environments and how these views affect its integration. The central research question is: How do healthcare professionals view the fit of guided iCBT in their settings, and how do these perceptions shape its implementation?

### Theoretical approach

The present study treats fit as a dynamic factor that shapes the extent to which the intervention integrates into the context, ultimately affecting the success of its implementation. We adopt a social constructivist perspective, asserting that fit is not an objective reality but is shaped by subjective perceptions. Different stakeholders may interpret and evaluate fit differently, influenced by their roles, experiences, and perspectives within the context.

Lau et al. (53) proposed a framework that identifies four key elements: the intervention itself, the individual and professional context, the organisational context, and external factors. The framework characterises the intervention in terms of aspects such as usability, complexity, cost-effectiveness, and security. Context is divided into three levels: the individual and professional level, which relates to healthcare providers’ attitudes, experience, and alignment with the intervention; the organisational level, involving internal aspects such as culture, resources, equipment, and management; and the external level, covering influences outside the organisation, such as policies, infrastructure, and economic conditions. This framework emphasises how interactions between interventions and these contextual layers shape implementation outcomes, underscoring the need for further study of these factors.

Consistent with the study aim, Lau et al.'s (53) framework was selected because of its explicit focus on fit between interventions and multiple contextual levels. This focus aligned closely with our interest in healthcare professionals’ perceptions of the suitability of guided iCBT across professional, organisational, and wider healthcare contexts.

## Methods

### Study design

A qualitative research approach was used to thoroughly explore participants’ perspectives and understanding of guided iCBT within their current clinical contexts. To support this, a case study design was selected, allowing an in-depth examination of the contextual factors participants perceived as influencing implementation. Case study research investigates real-life, contemporary bounded systems (cases) over time, using detailed, in-depth data collected from multiple sources (54). This approach facilitates a comprehensive understanding of complex phenomena within their natural settings. Including three distinct clinical settings introduced contextual variation, aiming to improve the overall understanding of the contextual influences on the implementation process.

### Study participants

Participants were purposively selected from three service units offering guided iCBT. Eligible participants included therapists delivering guided iCBT, therapists without experience of guided iCBT working elsewhere in the organisations, leaders at different organisational levels, and support personnel involved in implementation, supervision, technical support, or service development. The aim was to recruit individuals with varied professional roles and levels of experience with guided iCBT in order to capture both operational and strategic perspectives on implementation. Team leaders invited all eligible personnel via email.

The exact number of invited participants is unknown because team leaders distributed invitations rather than sending them directly to therapists. Most staff working in the participating iCBT clinics took part in the study, and leaders from all organisational levels relevant to the implementation effort were represented. Most participants were recruited through purposive sampling, while additional support personnel were identified through snowball sampling.

Recruiting therapists without experience with guided iCBT proved more challenging because access depended on support from local leaders. One participant withdrew for personal reasons, one did not attend without explanation, one leader declined participation because of time constraints and competing work demands, and two leaders did not respond to requests to facilitate recruitment of non-iCBT therapists. Because participation was voluntary and recruitment was mediated through team leaders, we could not assess whether non-participants differed systematically from participants in professional background, attitudes toward guided iCBT, or implementation experience.

The study included 31 participants: 15 therapists, 11 leaders, and 5 support staff. The sample was predominantly psychologists, most of whom had experience with guided iCBT. Most participants were female, and slightly more than half reported current or prior involvement in delivering guided iCBT. In addition to psychologists, the sample included nurses, medical doctors, and individuals from other professional backgrounds. The distribution of roles, gender, and experience with guided iCBT is summarised in Table 1.

**Table 1:** Participant characteristics by profession, gender, and iCBT experience (*N* = 31).

Profession (*n*)	Female (*n*)	Male (*n*)	Experience with iCBT^a^ (*n*)
Psychologist (*n* = 20)	11/20	9/20	15/20
Nurse (*n* = 6)	4/6	2/6	2/6
Medical doctor (*n* = 3)	2/3	1/3	0/3
Other (*n* = 2)	1/2	1/2	0/2
Total (*N* = 31)	18	13	17

a Experience with guided iCBT was defined as having previously or currently delivered at least one treatment course of internet-based cognitive behavioural therapy (iCBT) with therapist support, using the eCoping platform.

### Data generation

We employed three sources for data collection: background interviews conducted by phone and digitally, digital focus-group and individual interviews, and in-person observations.

### Background interviews

BS contributed to three individual phone interviews with leaders of the specialised mental health centres housing the eCoping clinics. These discussions focused on clinic organisation, including staff roles, daily operations, workflows, and implementation activities. Field notes were taken and returned to participants for comments and corrections, and follow-up interviews with leaders who later also participated in individual interviews.

### Focus group and individual interviews

Semi-structured interviews with open-ended questions were used to explore healthcare professionals’ experiences implementing guided iCBT. A total of 22 interviews, six focus group interviews, and 16 individual interviews were conducted by BS from January to June 2022.

The interview guide (Supplementary Appendix A), developed collaboratively by the authors, covered participants’ thoughts and experiences with guided iCBT. Due to COVID-related travel restrictions, most interviews were conducted via Microsoft Teams video calls, with two individual interviews conducted by phone at participants’ request. Participants participated from work or from their home office, with no non-participants present.

All interviews were conducted in Norwegian, recorded, and transcribed verbatim by BS Relevant quotations were later translated into English. Two transcripts were returned to participants upon request. Field notes were taken immediately after each interview to capture nonverbal cues, contextual details, and reflections on how the interviewer's role and behaviour might have influenced the interactions, ensuring a reflexive approach to data collection.

Focus group interviews, averaging 66 min (range: 51–80), were conducted with therapists to foster dynamic discussions among participants and minimise interviewer influence. Individual interviews, averaging 38 min (range: 25–50), were conducted with leaders and support staff to provide a more private setting for open dialogue. All interviews were included in the analysis.

### Observations

Participatory observations were conducted to capture contextual data that video interviews might have missed, including work procedures, office spaces, and colleague interactions. The first author spent one full day in each of the three clinics, attending treatment and clinic meetings, observing therapists delivering guided iCBT, and conversing with therapists and leaders about their work. She also observed two two-day collaborative meetings, one for clinic team leaders and another for head psychiatric leaders and their subordinate managers. In total, the data include seven days of participatory observations.

### Reflexivity

Reflexivity entails critically examining the knowledge produced and the researcher's role in its creation (55). From a constructivist perspective, research outcomes are shaped by the researcher's preconceptions (56). The first author (BS) had previously worked in one of the participating clinics, ending seven years before the study. She therefore knew some of the staff professionally. BS is a licensed psychologist specialising in adult clinical psychology and has 12 years of experience in public health care. At the time of the study, she was a PhD candidate at a Norwegian university. She had an interest in, and a positive attitude toward, guided iCBT, and therefore paid particular attention to how her assumptions and professional background might influence the research process. While familiarity with some participants may have influenced interview interactions, it may also have facilitated openness and richer accounts. Many participants were not previously known to BS, and interview questions encouraged discussion of both positive and negative experiences with guided iCBT. To support reflexive engagement with the data, BS documented preconceptions before data collection and maintained a reflexive journal throughout the study. BS also regularly discussed emerging interpretations with co-authors, who brought different professional and disciplinary perspectives to the analysis. These discussions supported ongoing critical reflection on the research process and its impact on knowledge production.

### Study context

The study was conducted in three specialised outpatient mental health clinics in Norway, which are part of the publicly funded specialist healthcare system. Access to regular outpatient treatment typically requires a referral from a general practitioner (GP), and patients pay a nominal fee per session. For guided iCBT, patients may self-refer in some clinics, and there is no fee for written communication with the therapist. However, a small co-payment is required for the initial assessment, the final session, and any additional contact with the therapist during the treatment period, such as phone calls or in-person or digital meetings. A more detailed description of the Norwegian mental healthcare system is provided elsewhere (57).

### Cases

The three cases examined are clinics within specialised mental health centres, known as “eCoping” (Norwegian: eMeistring), that provide guided iCBT. The service changed its name in spring 2024 and is currently known as eTreatment (Norwegian: eBehandling); however, the term eCoping is used throughout this article, as this was the name at the time of the study. One eCoping clinic has delivered guided iCBT in routine care since 2013, while the other two were established in 2014 and 2017. At the time of data collection, 15 eCoping clinics were operational across Norway as part of an ongoing national implementation effort. These clinics offer guided iCBT for social anxiety, panic disorder, and depression (58–60). Patients undergo an assessment interview before starting the 14-week programme, which comprises 8–9 modules and weekly written guidance from licensed health professionals. The programme is delivered through a secure platform, where patients also submit symptom reports via digital questionnaires. In this study, “eCoping” refers to the clinic, while “guided iCBT” denotes the treatment programme, including interviews, digital modules, and written therapist guidance (23).

Table 2 outlines the key characteristics of each clinic. Notably, while two clinics shared a workspace for therapists, Clinic 1 did not; its staff worked from separate offices. Clinic 1 previously shared an electronic platform with another clinic, requiring quarterly visits for updates. COVID-19 travel restrictions hindered these updates, limiting their capacity to provide guided iCBT. At the time of the interviews, Clinic 1 had been using its own platform for less than a year and was gradually re-engaging more participants. Clinic 2 held a unique position among the three clinics, often referred to as the “mother ship.” This clinic was responsible for tech support and programme development, and staff from other clinics turned to it for guidance due to its extensive experience. It was also the only clinic organised in its own department. Clinic 3 was unique in that it did not allow self-referrals and did not conduct its own assessments. Instead, it received patients whom other parts of the organisation had already assessed. This meant that Clinic 3 did not control the waiting time for assessments to the same extent as the other clinics.

**Table 2:** Clinic characteristics.

Feature	Clinic 1	Clinic 2	Clinic 3
Years operating	5	9	8
Organisation/roles	Team (short-term dept.); flexible roles^a^	Dedicated dept.; fixed 50% roles	Team across depts.; fixed 20–40% roles
Referral source	GP, therapists, self-referral	GP, therapists, self-referral	GP, therapists
Assessment on-site	Yes	Yes	No
Implementation goal	Increase referrals	Increase treatments & assessments	None defined
Unique features	Separate locations; new platform (1 yr)	“Mother ship” (development, tech support)	No self-referrals

a Therapists worked approximately 20% with eCoping and 80% face-to-face.

### Analysis

The three clinics studied are located in Norway, providing a shared contextual foundation. However, participants may perceive and highlight different aspects of their context, and certain contextual elements likely differ across clinics. To capture these dynamics, each clinic was initially analysed separately to identify contextual similarities, differences, and site-specific patterns. The findings from the three clinics were subsequently compared to identify both shared patterns and contextual variation. These insights were integrated into broader themes organised around Lau et al.'s (53) framework while retaining attention to how perceptions of fit differed across settings.

With support from the other authors, BS analysed interview transcripts, observation notes and background interview material. Coding was conducted in Microsoft Word and followed a stepwise deductive-inductive approach (61), informed by principles of reflexive thematic analysis (55). The analysis began with repeated readings of the material to support familiarisation and generate initial, data-driven codes grounded in participants’ accounts and observational data. These codes were then developed into preliminary categories, which were iteratively compared with and refined through Lau et al.'s (53) contextual fit framework and its key domains (Supplementary Appendix B). Throughout the process, the analysis moved back and forth between the empirical material, emerging interpretations, and the conceptual framework, allowing categories to be refined while remaining grounded in participants’ experiences.

Coding decisions, analytic reflections, and successive revisions of categories were documented throughout the process, providing a record of the development of the analysis. Emerging interpretations, codes, and themes were regularly discussed among the authors. Differences in interpretation occasionally arose regarding the categorisation and meaning of codes but were resolved through discussion and renewed examination of the original material. Organising the findings around Lau et al.'s framework and its domains (Supplementary Appendix B) provided a systematic, transparent structure for interpretation while facilitating comparisons with previous studies using the same conceptual approach.

Adopting a reflexive approach (55), we did not frame data adequacy in terms of saturation. Instead, meaning was understood as constructed through interpretation, and thematic sufficiency was treated as an analytic judgment. Themes were considered sufficiently developed when they demonstrated coherence, depth, and explanatory value in relation to the research question, based on iterative engagement with the data and recurring patterns across interviews and observations.

### Trustworthiness

Trustworthiness was ensured by attending to credibility, dependability, reflexivity, and transferability in the design, data collection, and analysis (62). Credibility was strengthened by using multiple data sources, including individual interviews, focus groups, background interviews, and observations, thereby enabling triangulation of findings. Interpretations and coding decisions were also discussed among all authors throughout the analytic process. In addition, two transcripts were returned to participants upon request; however, no comments or corrections were received. Dependability was supported by a systematic and transparent analytic approach, in which coding was iteratively developed and checked against the data. Reflexivity was maintained through continuous reflection on the researcher's role and assumptions (see Section “Reflexivity”). Transferability was facilitated by providing detailed descriptions of the study context, participants, and the three clinical cases, including variation in organisational structures and implementation conditions, allowing readers to assess the relevance of the findings to other settings.

### Ethical considerations

Participants received standardised written and oral information about the study and the researchers’ role before the interviews and observations. All participants signed a consent form. The study was approved by the university's data protection system and presented to the Regional Committees for Medical and Health Research Ethics (case number 396856). No further ethical approval was required.

## Findings

The findings are structured according to the conceptual framework proposed by Lau et al. (53), with the elements—encompassing the intervention itself, individual and professional context, organisational context, and broader external context—guiding the analysis and serving as thematic lenses through which the findings were interpreted and presented.

### The intervention

When describing the intervention, some participants regarded the treatment outcomes in guided iCBT as comparable to those of face-to-face therapy for certain patients, highlighting its capacity to treat a larger number of individuals and the positive feedback it received from both therapists and patients. Others, however, noted that, although underpinned by strong evidence, it often failed to produce meaningful outcomes in routine clinical practice.

The treatment programme's interface was found to be inadequate. It was text-heavy, underutilised the technology's potential, and featured an outdated and cumbersome portal. Conversations during participatory observations revealed that even professionals working with guided iCBT were reluctant to promote it because of its poor interface. To improve engagement and accessibility for a broader range of patients, participants suggested incorporating more interactive elements, such as videos and audio content.

“I thought it was difficult to get through […] the modules were very long, there was a lot of text, and the way it was presented, how you navigated through the module, I found it a bit difficult and unengaging, making it easy to lose focus. Especially when you're used to modern game development that provides a lot of feedback […] this felt quite outdated in comparison.”

(Leader and therapist 28, Clinic 3)

This quote captured a sentiment shared by several participants, emphasising that the programme should have been designed to foster communication between the patient and the intervention itself, in addition to the interaction with the therapist. The limited availability of diagnosis-specific treatment programmes, which covered only three conditions, also constrained the broader applicability of guided iCBT.

Participants noted that the rigid structure of the existing IT system impeded efforts to enhance or customise the treatment programmes, making it difficult to adapt to emerging best practices. They also described the treatment programmes as poorly integrated with other IT systems, which complicates their use within the broader healthcare infrastructure and creates additional work for therapists. Participants further noted that the nature of guided iCBT was often poorly understood, leading to misaligned expectations among both providers and patients.

“In order for people to understand what it is, we have to spend a lot of time explaining what it actually involves.”

(Support staff 14 with previous experience as an iCBT therapist, Clinic 2)

Uncertainty about what guided iCBT entails was also evident at the specialised mental health centre where it was being implemented. Participatory observations revealed that a leader recently stated they did not consider working with guided iCBT to be actual clinical work. This surprised the informants, as the treatment had been provided at the specialised mental health centre for several years, and they expected the leader to be more knowledgeable about it.

The three clinics held differing views on the intervention, which could affect its implementation. Participants from one clinic had a notably more positive view of the intervention's user-friendliness than those from the other clinics. This may facilitate smoother adoption and greater enthusiasm among staff. In another clinic, there was a distinct emphasis on the challenges of scaling the intervention, a concern not raised in the other clinics. This focus on scaling issues may benefit all clinics, as scaling is a prerequisite for successful implementation. Informants from a third clinic reported that healthcare personnel viewed guided iCBT as a second-rate treatment and that outcomes were poorer than those observed in research studies. This perception could undermine the credibility and acceptance of guided iCBT among healthcare personnel, thereby hindering its implementation in this clinic.

### The professional contextual level

When participants explained the perceived usefulness of guided iCBT, they noted differing views among therapists. They explained that those trained in Cognitive Behavioural Therapy (CBT), as well as younger or more open therapists, tended to have a more positive outlook on this treatment method. To build confidence in guided iCBT, therapists needed strong research evidence, personal experience, and patient success stories. One of the leaders sums up what many participants believed:

“It was important that research was in place. Being able to say that it is effective is really the most decisive factor. However, it was also very positive to have personal experience as a therapist and to see firsthand how patients benefit from it.”

(Leader 2 with experience as an iCBT therapist, Clinic 1)

Firsthand experience with the intervention was considered essential to shaping therapists’ perceptions of and acceptance of guided iCBT. Initially, many therapists were sceptical about its effectiveness. However, after personally engaging with the treatment, their attitudes changed markedly. This direct experience improved their confidence and encouraged them to incorporate the treatment into their clinical practice.

Some therapists felt that iCBT reduced the personal connection with their patients, yet they appreciated the increased frequency of contact compared to traditional face-to-face sessions. Participants observed that certain therapists, especially those with a psychodynamic background, remained sceptical of guided iCBT's validity as a treatment. One participant cited Max Planck (63, pp 33-34), saying, “*A new scientific truth does not triumph by convincing its opponents and making them see the light, but rather because its opponents eventually die, and a new generation grows up that is familiar with it.”* Therapists recognised that resistance to change posed a significant obstacle to the wider adoption of guided iCBT. However, even therapists who were well-versed in the benefits of guided iCBT sometimes found it difficult to believe that it could be as effective as face-to-face therapy. Participants also noted that other treatments often produced faster results and better outcomes, making it harder to justify opting for guided iCBT.

“What has been difficult for the therapists, which I understand, is the thought: ‘We already have the gold standard here, a 4-day treatment, so why not offer that right away?’”

(Leader 30, Clinic 3)

This leader recognises that other effective treatment methods are available for patients, but these approaches require more time from the therapist. Guided iCBT was viewed as beneficial because it requires minimal therapist time per patient. Additionally, some leaders emphasised the importance of offering a range of treatment options to ensure flexibility in addressing patient needs.

A blended approach that combined elements of iCBT and face-to-face therapy was favoured by some participants. One participant, who expressed a critical stance toward guided iCBT, described a blended approach as essential for ensuring responsible patient care and enabling continuous clinical judgment about which aspects of treatment could be conducted digitally while maintaining patient safety. Participants with a more favourable view noted that integrating face-to-face interactions could enhance patient motivation and improve treatment outcomes. Some even characterised blended therapy as representing the future of psychological treatment.

“I imagine that in the future we’ll have much more mixed care, where in certain phases there's face-to-face contact, and then it transitions to digital follow-up at home, before being brought back in again for face-to-face sessions.”

(Leader 9, Clinic 1)

Participants’ descriptions of the professional level also varied by clinic affiliation. In one clinic, CBT therapists held a positive view of guided iCBT, whereas influential therapists with a psychodynamic approach were sceptical about its suitability as a treatment method. These influential stakeholders could hinder implementation by fostering resistance and scepticism among other staff members, thereby affecting overall acceptance and integration of the intervention.

Participants from another clinic reported that the intervention had gained greater acceptance and credibility in recent years and that they recognised an opportunity to exercise professional autonomy by adapting the treatment in response to patient interactions, both of which can facilitate implementation. Participants at a third clinic reported that many therapists faced a professional dilemma about offering a treatment they perceived as second-rate. Although therapists who used iCBT in their work emphasised the flexibility it provided, the perception of the treatment as inferior could potentially hinder its implementation. Informants from the three clinics demonstrated diverse perceptions of the professional context, potentially leading to varied effects on intervention implementation across settings.

### The organisational contextual level

Participants underscored the importance of organisational factors in implementing guided iCBT, noting its distinct approach to treatment. This distinctiveness often left clinics unprepared for its integration. Despite receiving explicit support from leadership, practical backing frequently fell short. Participants highlighted the absence of clear targets, implementation plans, and dedicated resources needed to manage the new tasks associated with iCBT. This gap in practical support underscored the challenges healthcare organisations faced in adapting to the unique demands of guided iCBT.

“At times, the research and development department has provided valuable support, but this assistance should have been available much earlier. (…) eCoping itself took responsibility for spreading this with the resources available. I see that the leaders in eCoping have spent an enormous amount of time on agreements and things that I think should have been handled by legal experts, not frustrated therapists who don't have the expertise for it.”

(Leader 11, Clinic 2)

This leader clarifies the organisational challenges and lack of preparedness in implementing guided iCBT. The leader notes that although the research and development department eventually provided valuable support, it was not timely enough to be fully effective. The responsibility assigned to the clinic for implementation management, coupled with leaders spending excessive time on legal matters beyond their expertise, underscores the need for improved organisational planning and the appropriate delegation of tasks.

Observations revealed that leaders of eCoping clinics were required to undertake additional secretarial duties because they had to report to both researchers and other leaders, an unplanned task and consequently fell to them. They did not have a job description and were unsure whether their superiors were even aware of all the work they were undertaking. This highlights the organisation's and its leaders’ shortcomings in providing timely support, strategic planning, and effective delegation during implementation. Participants from all clinics reported that the organisation was not sufficiently prepared. Some pointed to gaps in the organisational model, such as the need for staff to take on new responsibilities, as noted by the leader above. Others emphasised cultural factors, including staff scepticism toward the treatment.

“There is indeed a very significant cultural barrier, both in relation to patients, practitioners, and referrers. We often encounter the attitude that it is better to talk face-to-face.”

(Leader 9, Clinic 1)

The leader acknowledges a distinct culture that prefers face-to-face interactions, both within the organisation and externally. This preference is also evident at the professional level, as previously demonstrated, and in the external context, which will be elaborated upon later. This recognition is crucial because it underscores the multifaceted nature of the cultural barriers encountered.

The clinic's organisational structure and priorities may hinder the implementation of guided iCBT. For instance, requiring referrals from colleagues to initiate treatment within a dedicated team adds an extra step that can act as a barrier. Another obstacle was the lack of protected time for therapists to engage effectively with guided iCBT. The organisation also frequently allocated resources to competing treatment approaches, which detracted from the focus on guided iCBT. Patients in specialised care were described as more seriously ill than before and often perceived as too complex for therapy, especially guided iCBT. This raised concerns among professionals about the intervention's suitability in this setting. Some argued that guided iCBT was less helpful for the complex and multifaceted needs of these patients. As a result, several professionals suggested that the programme might be better suited for primary care settings, where they believed patient cases are generally less severe.

“Since the target group is more in the mild/moderate range, there are likely some who question whether it is the right priority for specialist healthcare services to be involved in this.”

(Leader 29, Clinic 3)

This leader emphasises the importance of considering the clinical context when implementing digital interventions to ensure alignment with the needs of the target patient population. Taken together, participants highlighted several organisational factors that influenced the implementation of guided iCBT. These included organisational readiness, such as the availability of clear targets, implementation plans, sufficient resources, and protected time for therapists. Institutional factors, including how clinics were organised within the larger system, their locations, and competing treatment priorities, also shaped the integration of guided iCBT into practice. In addition, participants reported perceptions of patient complexity, with many describing patients in specialised care as too complex for guided iCBT, thereby influencing therapists’ willingness to adopt the intervention.

Organisational perceptions showed minor variations across the three clinics. One clinic had strong leadership support for guided iCBT, with well-defined treatment goals and a strategic position within departments familiar with short-term treatments and open to new methods. Despite favourable conditions for implementation, a lack of dedicated time often led therapists to fall back on usual routines in busy settings. Another clinic faced significant organisational challenges, including heavy assessment workloads that led to delays, additional non-clinical duties, and limited office space, all of which hindered effective staff collaboration. Some efforts to recruit highly skilled professionals aimed to enhance guided iCBT's profile, but the absence of a self-referral option likely limited patient access. Additionally, limited leadership support for implementation tasks complicated embedding guided iCBT into daily practice. Overall, staff views on the organisational context varied between clinics, likely influencing the implementation process.

### The external contextual level

Participants highlighted various external factors outside the health organisation that influence the healthcare landscape and the adoption of digital therapies. Guided iCBT aligned well with the broader trend of increased digitalisation in society. The growing number of patients and a shortage of therapists also made guided iCBT suitable, as it allowed each therapist to treat more patients. Participants from all three clinics emphasised the importance of research on guided iCBT, noting that its research-based foundation was essential to practitioner confidence. Their comments indicated a general adherence to the prevailing research paradigm, in which evidence-based practice is a key driver of therapeutic adoption.

“Research shows that guided iCBT was comparable to traditional face-to-face therapy. However, our results were poorer when we checked… Therapists are primarily concerned with ensuring that the treatments they provide are effective.”

(Leader 30, Clinic 3)

This statement highlights a perceived gap between the broader evidence base and local clinical outcomes, suggesting that contextual or implementation factors may limit the effectiveness of guided iCBT in practice. It also underscores that, while the dominant research paradigm facilitates the adoption of guided iCBT, its continued use depends on demonstrable benefits in specific service contexts.

Despite most research evidence demonstrating the effectiveness of guided iCBT, the perception of therapy as an in-person interaction remained a significant barrier to its acceptance. As we have seen, this persists across all levels of the context. Both patients and therapists frequently regarded traditional face-to-face therapy not only as the most effective form of treatment but also as the standard against which other interventions were measured.

“Many people come with a diagnosis of depression or anxiety and prefer face-to-face treatment. That is also the norm for what therapy is in society. “Fortunately,” the pandemic helped to loosen this up a bit, but that is still the norm, right?”

(iCBT therapist 18, Clinic 2)

“I know what therapy is, that's when I sit and talk to someone.”

(Support staff 14, reporting patients’ views, Clinic 2)

Participants working with guided iCBT argued that shifting this therapeutic paradigm required targeted efforts to educate both patients and professionals. Across all three clinics, participants emphasised the importance of information and marketing for professionals and potential patients. They believed that increasing awareness and understanding of guided iCBT's potential could lead to wider acceptance.

“I don't think patients are well informed about what therapy is (…) Are we good enough at communicating the benefits of this? I mean, it helps. We create brochures, post about it here and there, and it's on our websites, but I wish there were a national campaign.”

(Support staff 31, Clinic 3)

The clinics also showed some differences in how informants viewed the external environment. One clinic prioritised strict clinical pathways for assessment and treatment, with specialists wielding significant authority in determining appropriate approaches. Many specialists were regarded as sceptical of guided iCBT, which could pose a barrier to its adoption. Another clinic's participants highlighted that the absence of national strategies and inclusion in clinical guidelines hindered implementation efforts. They also noted that societal factors have led to patients in specialised healthcare becoming more severely ill in recent years, with many now too complex to benefit from therapy. A third clinic was unique in identifying the exclusion of guided iCBT from patient activity metrics as an external barrier to implementation. These differing perceptions of the external context likely influence implementation outcomes across the clinics.

## Discussion

This study examined healthcare professionals’ experiences and perceptions of implementing guided internet-based cognitive behavioural therapy (iCBT) in specialised mental health services, using Lau et al.'s (53) framework, which was selected because of its explicit focus on fit between interventions and multiple contextual levels. The findings illustrate the complex interplay among intervention characteristics, professional attitudes, organisational conditions, and broader contextual influences that shape the perceived fit and adoption of guided iCBT.

### The intervention: complexities

The findings highlight key challenges in the technological design and interface of the guided iCBT intervention studied, particularly its limited usability, adaptability, and integration with clinical systems. These issues, widely noted in prior research (64, 65), undermine its perceived value and relative advantage (26, 66). Poor integration with existing IT systems aligns with observations by Nilsen et al. (67), who note that limited involvement and insufficient integration of the IT department can significantly hinder the implementation of e-health technologies and even give rise to resistance among key actors. Participants in the current study described the intervention as outdated and inflexible—features that hinder local adaptation and are known barriers to implementation (68). Despite its strong evidence base, the intervention's complexity, lack of clarity, and poor interoperability with existing IT systems reflect broader concerns in the literature about the adoption of digital interventions in routine care (33, 68, 69).

### Professional context: therapist attitudes and cognitive barriers

The findings indicate that therapists’ perceptions of guided iCBT vary by professional background and experience, reflecting how well it aligns—or fits—with their clinical values and practices. Consistent with implementation research (70–73), our findings suggest that attitudes shaped by identity, digital literacy, and emotional responses are crucial, though acceptance alone does not guarantee use (74). This gap underscores the importance of contextual fit: When guided iCBT conflicts with therapists’ conceptions of “real” therapy, especially among non-CBT practitioners, it is viewed as less legitimate. The present study finds that non-CBT therapists are more resistant to guided iCBT, contrasting with earlier research that reports no significant differences in attitudes between CBT and non-CBT therapists (75). Moreover, our study shows that younger therapists express more positive attitudes, possibly due to greater familiarity with and openness to digital technologies, as suggested by Estrela et al. (76). This is further supported by Mason and Woodward (77), who emphasise the importance of digital literacy and comfort with technology in adopting digital interventions.

The findings further support Greenhalgh's (26) perspective that cognitive, emotional, and professional factors influence how an intervention's feasibility is perceived. Therapists find it difficult to accept an intervention as legitimate or suitable if it conflicts with established professional norms.

### Organisational context: organisational readiness and patients’ characteristics

At the organisational level, participants noted limited preparedness to implement guided iCBT. Although leadership was supportive, essential enablers such as time, resources, and planning were insufficient, with some differences across clinics. Structural factors such as clinic location, integration within the broader system, and competing priorities influenced perceptions of how well the intervention fit and its adoption. These insights align with Greenhalgh's (26) concept of organisational innovativeness and other research highlighting the importance of structural readiness and supportive infrastructure for successful implementation (78, 79). Some participants questioned whether guided iCBT was suitable for specialised care, suggesting it might be more appropriate for primary care due to patient complexity. However, this view conflicts with evidence showing that symptom severity is similar in primary and specialised mental health settings (80). This ongoing perception illustrates how subjective judgments about patient suitability can impede implementation, even when objective data indicate otherwise.

### External context: cultural assumptions

Participants saw guided iCBT as well aligned with the evidence-based approach (81, 82) and broader digitalisation initiatives (11), which increased its credibility. This alignment, along with its ability to meet increasing patient demand, makes guided iCBT a scalable and practical solution, even though there is ongoing academic debate about whether a strong evidence base alone ensures therapeutic effectiveness (83).

However, iCBT's limited compatibility with entrenched cultural beliefs, especially the idea that therapy should be face-to-face, created a significant hurdle. This reflects ongoing challenges in establishing the legitimacy of digital mental health solutions (84) and the resistance encountered when innovations clash with established values (85). Participants highlighted that improving the perceived suitability (the “fit”) of guided iCBT may require shifting dominant therapeutic assumptions. This shift could be achieved through training and targeted education for patients, clinicians, and the public. Existing research shows that healthcare professionals’ attitudes can be positively shaped through information, training, and practical demonstrations (75, 86). Effective approaches might include involving local opinion leaders (87), distributing informational materials (88), conducting outreach visits (89), and offering structured training to enhance therapists’ confidence (77). Additionally, embedding digital health into early professional education may promote a more lasting sense of fit and acceptance (90, 91).

Despite its importance, the external context remains underexplored in implementation research (33, 92, 93). Moore and Evans (94) critique the field's predominant focus on individual-level determinants and advocate greater attention to systemic and contextual influences. Similarly, Pervin et al. (95) show that even when stakeholders are initially open to evidence-based interventions, contextual misalignment can impede adoption. These findings align with the present study's emphasis on the critical role of contextual fit in implementation success.

### The interplay of fit among the intervention, the professional, the organisational, and the external context

This study shows that the implementation of guided iCBT is shaped by the complex interplay between the intervention and three contextual levels: professional, organisational, and external. Therapists’ attitudes were influenced not only by the intervention's features but also by its perceived alignment with the dominant face-to-face paradigm of psychotherapy, which Lau et al. (53) classified as part of the external context. As a result, even when supported by a strong evidence base, guided iCBT was often viewed as misaligned with prevailing therapeutic norms, which in turn hindered its adoption. These findings suggest that perceived fit with the broader therapeutic paradigm may be as influential as empirical evidence in shaping implementation outcomes. Shifting this paradigm may ultimately prove more effective than targeting individual attitudes alone. However, such shifts are gradual and require challenging entrenched assumptions (96), generating innovative research (97), and reshaping professional perspectives over time (98).

Although the current therapeutic model in mental health services remains predominantly face-to-face, our findings indicate that blended therapy—defined as integrating in-person sessions with guided iCBT—may be more acceptable and contextually appropriate for many clinicians. Participants were more open to digital interventions when they were embedded within traditional therapeutic formats rather than positioned as replacements. This aligns with emerging research suggesting that blended therapy is gaining traction as a pragmatic approach to digital integration (99), preserving core elements of conventional therapy while leveraging the scalability and accessibility of digital tools (86). By bridging the gap between established clinical norms and technological innovation, blended therapy may enhance perceived fit and support more sustainable implementation within existing service structures.

Our findings also illustrate how the same intervention can be interpreted and contextualised differently across clinical settings. These interpretations were shaped by participants’ professional roles, local conditions, and organisational cultures, resulting in distinct interactions among contextual factors within each clinic. This variation directly influenced how well guided iCBT was perceived to fit existing practices. The importance of perceived fit for implementation success has been highlighted in previous research (81, 83), and our study builds on this by offering a more nuanced account of how fit is constructed in practice. Inspired by Mielke et al. (31), who adopt a constructivist approach and rapid ethnographic methods to explore contextual complexity, our analysis underscores the value of examining how the perceived fit of interventions is actively interpreted and negotiated within specific settings.

Taken together, our findings reinforce the view that successful implementation depends on an intervention's fit with professional, organisational, and external contexts. This aligns with Greenhalgh et al.'s (93) diffusion of innovations model, which emphasises the dynamic interplay among the innovation, its adopters, the organisation, the implementation process, and the wider system. In our study, participants construed the fit of guided iCBT in diverse ways, shaped by their clinical backgrounds and the environments in which they worked. When the intervention was perceived as contextually compatible, adoption was facilitated; when it was perceived as misaligned, implementation was hindered. A nuanced understanding of contextual fit—and strategies to strengthen it—will therefore be essential to the effective integration of digital interventions into routine mental healthcare.

### Limitations

The study has some limitations that should be acknowledged. First, although the analysis incorporated inductive coding, interpretation was ultimately guided by a single conceptual framework. While this approach provided a structured lens for interpretation, incorporating multiple analytical perspectives could have enriched the findings by offering a broader understanding. It is also possible that certain themes or nuances fell outside the scope of the chosen framework and thus may not have been fully captured in the analysis.

Second, while the study offers rich insights into the context described by healthcare professionals, it lacks quantitative data on actual adoption rates, limiting our ability to link perceived contextual fit to measurable implementation outcomes. Future research should integrate quantitative measures to systematically assess adoption levels, thereby providing a more comprehensive view of how the interplay between the intervention and different levels of context affects implementation.

Third, the interviews were conducted at a single point in time. This cross-sectional design limits insight into how perceptions of fit and implementation challenges may change over time. A longitudinal approach could have captured dynamic developments, such as increased familiarity with the intervention, shifting organisational priorities, or changes in support structures.

Fourth, the study was conducted in three specialised mental health clinics within a single national healthcare system. While the aim of qualitative research is not statistical generalisability but rather transferability (62), this context-specific focus may limit the applicability of the findings to other settings.

Fifth, recruitment through team leaders may have introduced self-selection effects, as individuals more interested in guided iCBT or implementation issues may have been more inclined to participate. Because team leaders distributed invitations and participation was voluntary, the total number invited was unknown. We therefore could not assess whether non-participants differed systematically from participants in professional background, attitudes towards guided iCBT, or implementation experience. This may have influenced the range of perspectives represented in the material.

Finally, it is important to recognise the roles of the researcher and participants in data collection. The interviewer's prior knowledge of the intervention and familiarity with the setting may have affected how questions were asked and how participants responded. Participants might have highlighted or minimised organisational issues based on their roles or relationships with the implementation. Although the study acknowledged the researcher's subjectivity as part of the qualitative approach and aimed to improve transparency and trustworthiness through reflexivity, it remains essential to acknowledge that such efforts cannot fully capture how positionality influences the research process.

### Conclusions and implications

Our qualitative research indicates that adoption of guided iCBT depends on how well it fits perceived needs and on the intricate interactions across three contextual layers: professional, organisational, and external. At the intervention level, a perceived gap between the robust evidence supporting guided iCBT and its outdated, inflexible format diminished its perceived usefulness. Clinicians’ backgrounds and therapeutic orientations shaped the extent to which the intervention aligned with professional norms. Limited organisational readiness and concerns about managing patient complexity in specialised settings raised questions about its suitability. Moreover, tensions between the research-focused emphasis on evidence and the prevailing therapeutic approach that favours in-person care further challenged its perceived legitimacy.

This study indicates that guided iCBT serves as a convergence between new digital methods and traditional therapeutic practices. Unless the complexities across contextual levels are better understood and addressed, efforts to improve fit may remain insufficient. The differences observed across clinics emphasise the importance of developing customised implementation strategies that account for local issues and perceptions.

This study indicates that, for effective implementation, guided iCBT must be designed to enhance its relevance and credibility among clinicians. Additionally, successful adoption depends on addressing clinicians’ attitudes, strengthening organisational readiness, and challenging existing views of what constitutes legitimate therapy.

An important insight from applying Lau et al.'s (53) framework is that guided iCBT does not fully align with the dominant therapeutic paradigm. Our findings suggest that guided iCBT may continue to face challenges in gaining legitimacy alongside traditional face-to-face and real-time approaches unless broader shifts in therapeutic thinking occur. A paradigm shift, driven by increasing the visibility of guided iCBT's effectiveness through rigorous research and by effectively communicating and integrating it in ways that resonate with professionals and align with their clinical values, will be crucial to moving guided iCBT from the margins to the mainstream of mental healthcare.

## Data Availability

The datasets generated and analysed during the current study are not publicly available to protect privacy and confidentiality in accordance with Article 24 of the Declaration of Helsinki. Requests to access the datasets should be directed to beatestandal@hotmail.com.
