# Supplementary material for: The perceived fit of guided iCBT in specialised mental healthcare services: a qualitative study of healthcare professionals’ perspectives
Source: Front Digit Health. 2026 Sep 9;8:1955151. doi: 10.3389/fdgth.2026.1955151 (PMC13597905; doi:10.3389/fdgth.2026.1955151)
Supplement: Supplementary file 1 [file Supplementaryfile1.docx]

# Appendix A:

## Interview guide

# 1. Can you tell me about your experiences with guided iCBT?

# a. Can you exemplify how it is used in your organisation?

# 2. What are your thoughts about providing guided iCBT?

# a. Is it a tool that can be used? How and for whom?

# 3. If we take for granted that guided iCBT should be used; What do you think it takes for this tool to be used and be recognised and considered equal to other treatment methods?

# a. What does it take in your daily work?

# b. What does it take in (name of workplace)?

# c. What does it take in (name of local health trust)?

# d. What does it take in (name of regional health trust)?

# e. What does it take on a national level?

# 4. Is there anything we haven’t talked about that you think is important regarding the introduction of guided iCBT?

# 5. Please mention three things that you think could make guided iCBT more widely used as a treatment method.

# Appendix B:

## Key themes related to the success or failure of the implementation of complex interventions

The table below outlines the 21 primary and 40 secondary themes identified in the conceptual framework developed by Lau et al. These themes were applied during the deductive phase of the analysis to categorise participant statements and guide the interpretation of implementation-related data.

|  | **Primary themes** | **Secondary themes** |
| --- | --- | --- |
| External context | Policy | Presence and form of policy |
|  |  | Presence of stated goals and objectives |
|  |  | Fit with local or national agenda |
|  |  | Presence of regulatory framework |
|  |  | Presence of code of practice |
|  | Infrastructure |  |
|  | Economic and financing |  |
|  | Incentives | Financial awards |
|  |  | Non-financial awards |
|  | Dominant paradigm |  |
|  | Public awareness |  |
|  | Stakeholder buy-in |  |
|  | Technological advances |  |
| Organisation | Culture | Organisational planning and readiness |
|  |  | Leadership |
|  |  | Hierarchy structure |
|  | Process and systems |  |
|  | Relationships | Inter-professional |
|  |  | Professional and patients |
|  | Resources |  |
|  | Skill-mix | Clarity about responsibility/role |
|  |  | Division of labour |
|  | Involvement | Support from team members and management |
|  |  | Collaborative working |
|  |  | Shared vision |
| Professional | Role | Professionalism |
|  |  | Sense of self-efficacy |
|  |  | Peer influences |
|  |  | Authority/influence |
|  | Underlying philosophy of care | Personal style |
|  |  | Relationship between professional and patient |
|  | Attitudes to change | Attitudes and beliefs (general) |
|  |  | Motivation and priority |
|  |  | Prior experience |
|  |  | Workload/competing demands |
|  |  | Perception of time |
|  | Competencies |  |
| Intervention | Nature and characteristics | Complexity |
|  |  | Evidence of benefit |
|  |  | Applicability and relevance |
|  |  | Clarity |
|  |  | Costs |
|  |  | Cost-effectiveness |
|  |  | Practicality and utility |
|  |  | Adaptability |
|  |  | IT compatibility |
|  | Implementability | Complexity of implementation |
|  |  | Benefit/harm of implementation |
|  |  | Resources requirements |
|  | Safety and data privacy |  |

Source: Lau R, Stevenson F, Ong BN, Dziedzic K, Treweek S, Eldridge S, et al. Achieving

change in primary care: causes of the evidence to practice gap: systematic reviews of

reviews. Implement Sci. 2016;11:40. doi:10.1186/s13012-016-0396-4.

## Appendix C:

**Consolidated criteria for reporting qualitative studies (COREQ): 32-item checklist**

| **No. Item** | **Guide questions/description** | **Reported on Page #** |
| --- | --- | --- |
| **Domain 1: Research team and reﬂexivity** |  |  |
| *Personal Characteristics* |  |  |
| 1. Interviewer/facilitator | Which author/s conducted the interview or focus group? | 6 |
| 2. Credentials | What were the researcher’s credentials? E.g. PhD, MD | 7 |
| 3. Occupation | What was their occupation at the time of the study? | 7 |
| 4. Gender | Was the researcher male or female? | 7 |
| 5. Experience and training | What experience or training did the researcher have? | 7 |
| *Relationship with participants* |  |  |
| 6. Relationship established | Was a relationship established prior to study commencement? | 7 |
| 7. Participant knowledge of the interviewer | What did the participants know about the researcher? e.g. personal goals, reasons for doing the research | 11 |
| 8. Interviewer characteristics | What characteristics were reported about the inter viewer/facilitator? e.g. Bias, assumptions, reasons and interests in the research topic | 7 |

| **Domain 2: study design** |  |  |
| --- | --- | --- |
| *Theoretical framework* |  |  |
| 9. Methodological orientation and Theory | What methodological orientation was stated to underpin the study? e.g. grounded theory, discourse analysis, ethnography, phenomenology, content analysis | 2-11 |
| *Participant selection* |  |  |
| 10. Sampling | How were participants selected? e.g. purposive, convenience, consecutive, snowball | 3-4 |
| 11. Method of approach | How were participants approached? e.g. face-to-face, telephone, mail, email | 3-4 |
| 12. Sample size | How many participants were in the study? | 4-5 |
| 13. Non-participation | How many people refused to participate or dropped out? Reasons? | 4 |
| *Setting* |  |  |
| 14. Setting of data collection | Where was the data collected? e.g. home, clinic, workplace | 5-6 |
| 15. Presence of non-participants | Was anyone else present besides the participants and researchers? | 6 |
| 16. Description of sample | What are the important characteristics of the sample? e.g. demographic data, date | 5, 8-9 |
| *Data collection* |  |  |
| 17. Interview guide | Were questions, prompts, guides provided by the authors? Was it pilot tested? | 6 |
| 18. Repeat interviews | Were repeat inter views carried out? If yes, how many? | 5 |
| 19. Audio/visual recording | Did the research use audio or visual recording to collect the data? | 6 |
| 20. Field notes | Were ﬁeld notes made during and/or after the interview or focus group? | 5-6 |
| 21. Duration | What was the duration of the inter views or focus group? | 6 |
| 22. Data saturation | Was data saturation discussed? | 10 |
| 23. Transcripts returned | Were transcripts returned to participants for comment and/or correction? | 11 |
| **Domain 3: analysis and ﬁndings** |  |  |
| *Data analysis* |  |  |
| 24. Number of data coders | How many data coders coded the data? | 10 |
| 25. Description of the coding tree | Did authors provide a description of the coding tree? | Appendix B |
| 26. Derivation of themes | Were themes identiﬁed in advance or derived from the data? | 10 |
| 27. Software | What software, if applicable, was used to manage the data? | 10 |
| 28. Participant checking | Did participants provide feedback on the ﬁndings? | NA |
| *Reporting* |  |  |
| 29. Quotations presented | Were participant quotations presented to illustrate the themes/ﬁndings? Was each quotation identiﬁed? e.g. participant number | 12-21 |
| 30. Data and ﬁndings consistent | Was there consistency between the data presented and the ﬁndings? | 11-21 |
| 31. Clarity of major themes | Were major themes clearly presented in the ﬁndings? | 11-21 |
| 32. Clarity of minor themes | Is there a description of diverse cases or discussion of minor themes? | NA |

Source: Tong A, Sainsbury P, Craig J. Consolidated criteria for reporting qualitative research

(COREQ): a 32-item checklist for interviews and focus groups. Int J Qual Health Care.

2007;19(6):349-357. doi:10.1093/intqhc/mzm042.
